# Familial Short QT Syndrome: Phenotypic Variability and Challenges in Risk Stratification

**DOI:** 10.3390/jcm15093461

**Published:** 2026-05-01

**Authors:** Paula Bouzón, Alberto Alen, María Salgado, Francisco González-Urbistondo, Lorena María Vega-Prado, Eliecer Coto, José Julián Rodríguez-Reguero, Juan Gomez, Barbara Fernández-Barrio, Pablo Avanzas, Rebeca Lorca

**Affiliations:** 1 Área del Corazón, Hospital Universitario Central Asturias, 33011 Oviedo, Spain; paulabouzoniglesias@gmail.com (P.B.); albertoalen@usal.es (A.A.); msalgadobarq@gmail.com (M.S.); frangonzalez_95@hotmail.com (F.G.-U.); josejucasa@yahoo.es (J.J.R.-R.); 2 Unidad de Cardiopatías Familiares, Área del Corazón y Departamento de Genética Molecular, Hospital Universitario Central Asturias, 33011 Oviedo, Spain; lorenamaria.vega@sespa.es (L.M.V.-P.); eliecer.coto@sespa.es (E.C.); juan.gomezde@sespa.es (J.G.); barfeba@gmail.com (B.F.-B.); 3 Instituto de Investigación Sanitaria del Principado de Asturias, ISPA, 33011 Oviedo, Spain; 4 Redes de Investigación Cooperativa Orientadas a Resultados en Salud (RICORs), 28029 Madrid, Spain; 5 Departamento de Medicina, Universidad de Oviedo, 33003 Oviedo, Spain; 6 Centro de Investigación Biomédica en Red de Enfermedades Cardiovasculares (CIBERCV), 28029 Madrid, Spain; 7 Departamento de Cardiología Pediátrica, Hospital Universitario Central de Asturias, 33011 Oviedo, Spain

**Keywords:** short QT syndrome, *KCNJ2*, channelopathy, sudden cardiac death, inherited arrhythmia, risk stratification

## Abstract

**Background:** Short QT syndrome (SQTS) is a rare inherited cardiac channelopathy associated with high risk of atrial and ventricular arrhythmias and sudden cardiac death (SCD). Data on its natural history, genotype–phenotype correlations, and risk stratification remain limited. We aimed to evaluate all families with a confirmed diagnosis of SQTS identified at our National Referral Center through a descriptive case series, thereby contributing additional real-world data on this rare condition. **Methods:** A retrospective review was conducted of all families evaluated for suspected SQTS between 2011 and 2025 at the Inherited Cardiac Diseases Unit. Diagnosis was based on 2022 ESC guidelines (QTc ≤320 ms or ≤360 ms plus supportive features), clinical evaluation, and genetic testing. Families meeting diagnostic criteria were included for detailed phenotypic and genotypic characterization and longitudinal follow-up. **Results:** Among all patients assessed, two families met the criteria for SQTS. One family with three phenotype-positive individuals was gene-elusive. This family had a history of SCD and the proband presented atrial fibrillation. The second family carried a pathogenic *KCNJ2* variant (p.Asp172Asn). However, only the proband fulfilled ECG criteria for SQTS (phenotype-positive) and there was no family history of SCD. No patients were treated with pharmacological therapy for QT prolongation. All affected individuals showed stable QT intervals (none <320 ms) and there were no malignant arrhythmic events during follow-up. **Conclusions:** These two families illustrate the wide phenotypic spectrum of SQTS and underscore the difficulty of risk stratification in asymptomatic individuals. The rarity of the disease, variable penetrance, and absence of robust prospective data hinder evidence-based management. Systematic registry participation and longitudinal studies are essential to refine risk prediction and therapeutic strategies.

## 1. Introduction

Short QT syndrome (SQTS) is an exceptionally rare inherited channelopathy characterized by abnormally short rate-corrected QT intervals (QTc ≤ 330–360 ms) [1] and an increased risk of atrial fibrillation, ventricular tachyarrhythmias, syncope, and sudden cardiac death (SCD) [1,2]. These manifestations can occur at any time from early infancy to old age [3]. The American Heart Association highlighted that SQTS is the newest recognized channelopathy associated with SCD, with prevalence likely underestimated due to underdiagnosis [4]. Since its first description in 2000 [5], SQTS has remained an exceptionally rare inherited channelopathy, with reported prevalence of 2.7 in 100,000 inhabitants or 0.02% to 0.1% [6] and only a limited number of published families and probands reported worldwide [3,7,8], mostly derived from small registries and observational series. Its true prevalence remains uncertain because of underdiagnosis, evolving diagnostic criteria, and referral bias [3,7,8].

Diagnosis relies on ECG findings, clinical presentation, and genetic testing. The Gollob score was first proposed in 2011 to assess diagnostic probability, but its reliability is debated [1,9]. According to the 2022 European Society of Cardiology (ESC) Guidelines [10], it is recommended (Class I, level C) that SQTS is diagnosed in the presence of a QTc ≤ 360 ms and one or more of the following: (a) pathogenic mutation, (b) family history of SQTS, or (c) survival from a ventricular fibrillation/tachycardia in the absence of heart disease [10]. SQTS should be considered (IIa, C) in the presence of a QTc ≤ 320 ms or QTc ≥ 320 ms and ≤360 ms and arrhythmic syncope, and may be considered (IIb, C) in the presence of a QTc ≥ 320 ms and ≤360 ms and a family history of sudden death at age < 40 years [10].

SQTS can present high lethality in all age groups, including the first months of life. Prognosis is variable. In early high-risk series, the cumulative risk of cardiac arrest has been reported to exceed 40% by the age of 40 years [11,12]. However, these estimates derive from early high-risk cohorts and may not be generalizable to contemporary or asymptomatic populations. Nonetheless, early recognition and intervention are critical to reduce morbidity and mortality. The ESC guidelines recommend implantable cardioverter-defibrillator (ICD) placement for secondary prevention [10]. However, primary prevention strategies in SQTS, including pharmacotherapy, remain controversial.

SQTS is most commonly inherited in an autosomal dominant manner, with variable penetrance and a male predominance. However, the diagnostic yield of genetic testing remains uncertain, and many cases may be genetically elusive. SQTS can result from pathogenic variants in potassium channel genes (*KCNH2, KCNQ1,* and *KCNJ2*) or anion exchanger *SLC4A3* [3,10]. According to ClinGen, only these four genes have sufficient evidence to be considered causative and should therefore be included in genetic testing panels for SQTS [13].

Data on the natural history, genotype–phenotype correlations, and clinical management—including risk stratification—remain limited due to the rarity of the condition and the scarcity of published case series. The aim of this study was to evaluate all families with a confirmed diagnosis of SQTS identified at our National Referral Center, describe their clinical and genetic characteristics, and discuss their management according to current ESC guidelines [10] through a descriptive case series, thereby contributing to the limited literature and expanding the scarce reported caseload of this rare condition.

## 2. Materials and Methods

### 2.1. Study Design and Population

A retrospective observational study was conducted, including all consecutive patients referred for genetic evaluation due to channelopathies, including possible SQTS, to the Inherited Cardiac Diseases Unit—a specialized cardiogenetic clinic accredited in 2011 as a National Reference Unit for the evaluation and management of inherited cardiovascular disorders—between January 2011 and January 2025.

This cohort represents a referral-based population enriched for suspected inherited arrhythmia syndromes and is therefore not intended to reflect population prevalence.

Diagnosis of SQTS was established if the individual met the 2022 ESC Guidelines diagnosis criteria, from Class I to IIb, level C recommendations [10]:

- QTc ≤ 320 ms (IIa, C).
- QTc ≤ 360 ms plus one or more of the following (I, C):
  ▪ Pathogenic variant (“mutation”);
  ▪ Family history of SQTS;
  ▪ Survival from a ventricular fibrillation/tachycardia episode without structural heart disease.
- QTc ≥ 320 ms and ≤360 plus one or more of the following:
  ▪ Arrhythmic syncope (IIa, C);
  ▪ Family history of sudden death before the age of 40 years (IIb, C).

Only families with an SQTS diagnosis based on the previous criteria were included. Isolated cases or borderline diagnoses were excluded.

Individuals were classified as phenotype-positive when they fulfilled diagnostic criteria for SQTS according to ESC guidelines [10].

Individuals carrying a pathogenic variant related to SQTS were classified as positive genotype.

Individuals carrying a pathogenic variant related to SQTS but without fulfilling clinical diagnostic criteria were classified as genotype-positive/phenotype-negative carriers.

### 2.2. Clinical Evaluation and Follow-Up

All individuals underwent a baseline comprehensive clinical assessment in our specialized cardiogenetic clinic, including detailed personal and family history, physical examination, 12-lead ECG, transthoracic echocardiogram (TTE), and, where indicated, Holter monitoring or exercise testing.

The QT interval was measured in lead II or V5 and corrected for heart rate primarily using Bazett’s formula, in line with current ESC guideline thresholds for SQTS diagnosis. Given the known limitations of Bazett correction at lower heart rates, QTc values were also recalculated using Fridericia, Framingham, and Hodges formulas to verify the consistency of phenotype classification.

All patients were followed in our specialized cardiogenetic clinic. ECGs and clinical evaluations were repeated annually. Device therapy and pharmacological management in our Unit were considered according to guidelines recommendations and tailored through shared decision-making with the patients [10].

### 2.3. Genetic Analysis

Blood samples were obtained from all patients who provided informed consent for genetic testing, using 9 mL EDTA-anticoagulated tubes. Genomic DNA was extracted from peripheral blood leukocytes using the standard salt extraction method, a simple and non-toxic protocol that yields high-quality DNA.

Genetic testing was performed on DNA samples from all referred patients. Next-generation sequencing (NGS) was conducted using a custom-designed panel of over 200 genes associated with inherited cardiovascular diseases, including arrhythmogenic syndromes, as previously reported by our group [14,15,16]. This panel was adopted as part of our routine, cost-effective diagnostic workflow for inherited cardiovascular disease evaluation rather than as an SQTS-specific assay. The panel included *KCNH2*, *KCNQ1*, *KCNJ2*, *CACNA1C*, *CACNA2D1*, and *SLC4A3* (added into the latest update, CARDIOv10, March 2025). Sequencing was performed using the Ion Torrent platform with semiconductor chip technology and the Ion GeneStudio S5 sequencer (Thermo Fisher Scientific, Waltham, MA, USA). Raw data were processed using Torrent Suite v5 software. Read alignment and variant calling were performed with Variant Caller (VC), and variant annotation—including population frequencies, functional predictions, and disease associations—was carried out using Ion Reporter (Thermo Fisher Scientific) and HD Genome One (DREAMgenics SL, Oviedo, Spain). The Integrative Genome Viewer (IGV, Broad Institute, Cambridge, MA, USA) was employed for in-depth coverage review, sequence quality assessment, and variant validation.

Identified variants with a minor allele frequency < 0.01 were evaluated by a multidisciplinary team comprising geneticists and cardiologists specialized in human genetics [16,17,18]. Variant classification followed the 2015 ACMG–AMP guidelines [19] and included categories of pathogenic (P), likely pathogenic (LP), variants of uncertain significance (VUS), and benign/likely benign. Only variants classified as P or LP were considered clinically relevant and are presented in the Results section. We did not include VUS in the final analysis because they do not provide diagnostic certainty for SQTS when QTc is ≤360 ms, and their inclusion would not have changed the composition of the cohort or the clinical conclusions of the study. Variant confirmation and segregation analysis were performed by Sanger sequencing using an ABI 3130XL sequencer (Thermo Fisher Scientific).

All participants provided written informed consent for the use of their genetic and clinical data for research purposes. All patients received pre-test genetic counseling, including information about the possibility of incidental or secondary findings [20], and signed informed consent accordingly. No incidental findings relevant to SQTS were reported in the present series. The study complied with institutional ethical standards and was approved by the Regional Ethics Committee (CEImPA 2025.109).

## 3. Results

**Figure 1:**
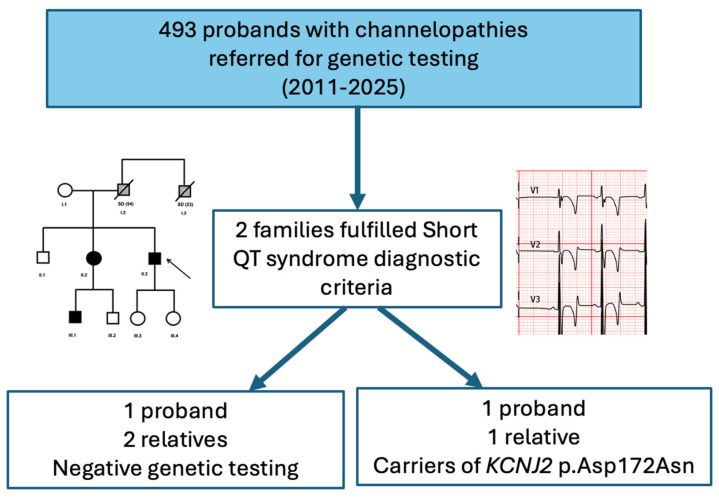
Flow chart of families with phenotype and/or genotype-positive SQTS.

From the 493 probands referred for genetic testing due to suspected channelopathies between 2011 and 2025, we identified two families that fulfilled diagnostic criteria for SQTS and were included for analysis (Figure 1). A summary of the main findings is presented in Table 1. Given the very small sample size and limited follow-up, the absence of arrhythmic events in this series should be interpreted with caution and does not allow any inference regarding arrhythmic risk.

Family 1: Genotype-negative SQTS

**Figure 2:**
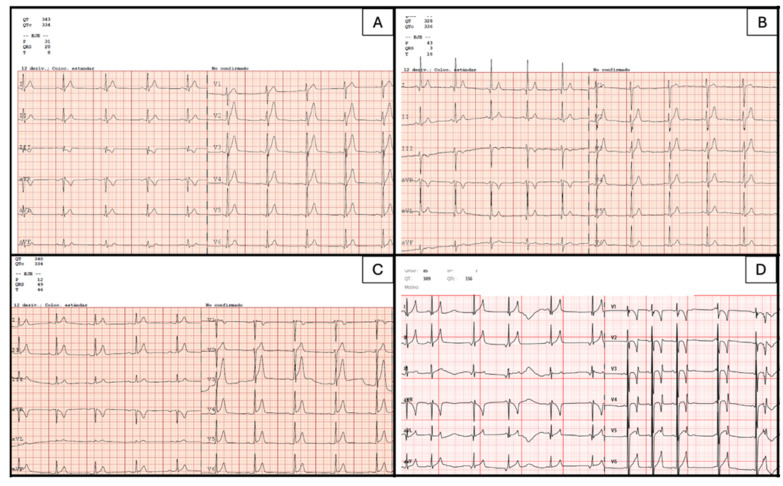
Electrocardiograms with QTc <360 msec. (**A**) Family 1. Proband (III.3). (**B**) Family 1. Proband’s sister (II.2). (**C**) Family 1. Proband’s nephew (III.1). (**D**) Family 2. Proband and carrier of *KCNJ2* p.Asp172Asn.

**Figure 3:**
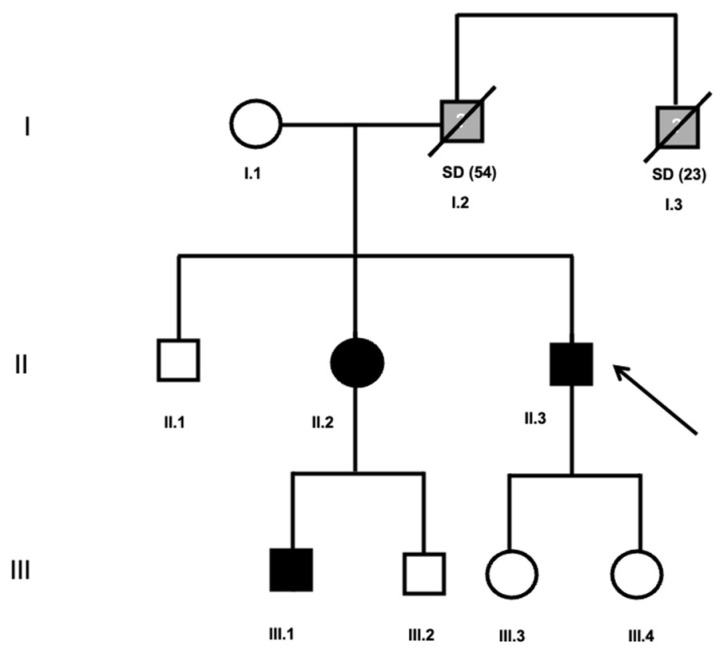
Family 1’s pedigree. SD, sudden death. Age of SD, in brackets. Symbols denote sex and disease status: box, male; circle, female; black darkened, short QT syndrome phenotype; grey darkened, unexpected SD; symbol clear, negative phenotype; ?, unknown phenotype; slashed, deceased; arrow, proband.

The index case was a 58-year-old man who presented with episodes of palpitations and dizziness, without syncope (II.3, Table 1). The ECG performed in January 2015 showed a QTc < 360 ms with tall, peaked, and symmetrical T waves (Figure 2A, Table 1). Coronary angiography demonstrated normal coronary arteries. Due to persistent symptoms, an implantable loop recorder was implanted, which revealed episodes of atrial fibrillation (AF) with rapid ventricular response. The patient had a family history of premature sudden cardiac death (father at 54 years and paternal uncle at 23 years, Figure 3). Therefore, given the QTc ≤ 360 ms plus family history of SCD before the age of 40 years old, the patient was diagnosed with SQTS (phenotype-positive). Genetic testing revealed no pathogenic variants. The case was presented at a multidisciplinary meeting, where management options were discussed. Although the patient did not meet a formal Class I indication for ICD implantation, the decision was influenced by the combination of a confirmed SQTS phenotype, documented atrial fibrillation, a markedly malignant family history of sudden death and the uncertainty surrounding individual risk prediction in SQTS. In this context, ICD implantation for primary prevention was considered in line with a Class IIb recommendation according to current ESC guidelines [10], and was ultimately performed after shared decision-making with the patient.

First-degree relatives underwent ECG screening. Two relatives showed a short QT interval, fulfilling criteria for SQTS diagnosis (phenotype-positive): a 60-year-old sister and her 42-year-old son (Figure 3). None had structural heart disease evaluated by a transthoracic echocardiogram. The nephew (III.1, Figure 3) was asymptomatic, with no history of syncope. His ECG performed in March 2021 showed a QTc < 360 ms (Figure 2C, Table 1) and Holter monitoring revealed no arrhythmic events. The sister (II.2, Figure 3) was also asymptomatic. Her ECG performed in June 2015 showed a QTc of <360 ms (Figure 2B, Table 1), and no additional findings in cardiological evaluation.

Genetic testing with the NGS panel was also performed in the two affected relatives, and, likewise, it did not identify any pathogenic variants. Following multidisciplinary discussion and shared decision-making, the patients were managed conservatively with close monitoring, without prophylactic pharmacological or device therapy.

During follow-up, none of the patients experienced any arrhythmic events, as confirmed by serial ECGs and Holter monitoring (with ICD monitoring for the proband). They remained entirely asymptomatic throughout the observation period.

Family 2: *KCNJ2*-related SQTS

**Figure 4:**
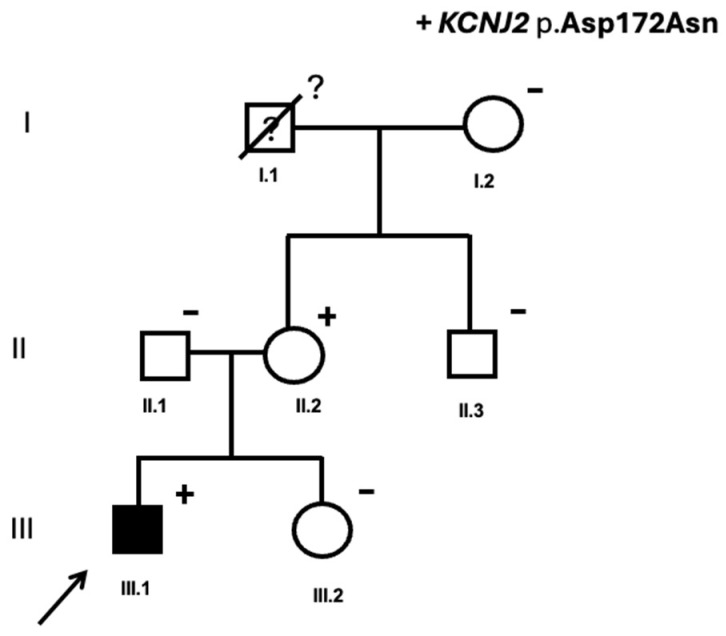
Family 2’s pedigree. Symbols denote sex and disease status: (+), *KCNJ2* p.Asp172Asn carriers; (−), non-carriers; (?), genetic status unknown; box, male; circle, female; black darkened, short QT syndrome phenotype; symbol clear, negative phenotype; ?, unknown phenotype; slashed, deceased; arrow, proband.

The index case was a 4-year-old boy (III.1, Figure 4) with an incidental diagnosis of short QT during follow-up for a small muscular ventricular septal defect. The ECG performed in March 2023 showed a QTc < 360 ms (Figure 2D). QTc values remained below diagnostic thresholds across all correction formulas, supporting a consistent phenotype-positive classification (Table 1). There was no family history of sudden cardiac death. Genetic analysis identified the heterozygous variant *KCNJ2* (NM_000891.3):c.514G>A p.(Asp172Asn). This variant, absent from population databases, has already been described by Priori et al. as a pathogenic variant for SQTS in a family with a unique ECG phenotype characterized by asymmetrical T waves, and a defect in the gene coding for the inwardly rectifying Kir2.1 (I(K1)) channel [21]. Functional studies from prior reports have shown that the D172N gain-of-function effect on the Kir2.1 channel increases the outward I(K1) current, leading to shortened effective refractory periods and increased arrhythmogenic susceptibility [22]. It was classified as pathogenic according to ACMG/AMP guidelines criteria [19] (Table 2).

The diagnosis of SQTS in the proband (phenotype-positive) was established on the basis of a pathogenic *KCNJ2* variant together with a short QT phenotype (QTc ≤ 360 ms), according to ESC criteria [10].

The patient remained asymptomatic with no history of syncope, and Holter monitoring showed no arrhythmic events during follow-up.

Evaluation of first-degree relatives revealed that the 39-year-old mother (II.2, Figure 4) carried the same *KCNJ2* pathogenic variant, with a QTc of 365 ms (Bazzet formula, October 2023). She was asymptomatic, with normal Holter monitoring, a normal exercise test and no structural heart disease evaluated by TTE. Because of the relatively low heart rate (51 bpm), QTc was also recalculated using Fridericia, Framingham, and Hodges formulas, yielding values of 375 ms, 369 ms, and 380 ms, respectively (Table 1). These results confirmed that she did not fulfill ECG criteria for SQTS and was, therefore, classified as a genotype-positive/phenotype-negative carrier.

All available first-degree relatives—the father, maternal grandmother, maternal uncle, and sister (Figure 4)—were evaluated and found to have normal QTc values and were non-carriers of the *KCNJ2* pathogenic variant. The maternal grandfather could have been a carrier of the variant. However, he died previously due to non-cardiac causes, without any documented cardiac history, and no genetic testing was performed. Therefore, it cannot be determined whether the variant in the mother arose de novo.

Following multidisciplinary discussion and shared decision-making, both carriers were managed conservatively with close monitoring, without prophylactic pharmacological or device therapy. An implantable loop recorder (ILR) was considered for the younger carrier, according to ESC guidelines [10].

## 4. Discussion

This national reference-center review identified two families with SQTS over a ten-year period, underscoring the rarity of this channelopathy and the diagnostic challenges it poses. Both families showed stable clinical courses during follow-up, with persistently short QT intervals and no documented malignant arrhythmic events. Nevertheless, management was individualized, and ICD implantation was performed in one proband due to a markedly adverse family history and shared decision-making. These observations illustrate the variable expressivity of SQTS within a limited sample and highlight the challenges in risk stratification. However, given the small number of families, these findings should be considered hypothesis-generating and not suitable for drawing definitive conclusions.

Diagnosis of SQTS has been challenging and somewhat controversial. Until formalized diagnostic criteria were proposed, there was no consensus regarding the appropriate QT interval cutoff for diagnosis, further complicated by the observation of short QTc intervals in apparently healthy individuals. Therefore, in 2011, Gollob et al. proposed SQTS Diagnostic Criteria as resulting in a high probability for SQTS when scoring ≥ four points and a low probability for SQTS with two or fewer points [1]. Currently, ESC guidelines provide objective QTc cutoffs of 320 ms or 360 ms, in addition to other phenotypic features [10].

In this study, both families fulfilled the ESC criteria for SQTS diagnosis. In Family 1, three individuals exhibited QTc < 360 ms along with a family history of sudden death, and the proband presented with early-onset atrial fibrillation. In Family 2, the proband presented with QTc < 360 ms, characteristic of T-wave morphology (tall, peaked, and narrow or symmetrical), and carried a pathogenic *KCNJ2* variant.

Genetics remains a challenging aspect of SQTS. Despite clear clinical diagnoses, many patients remain genetically elusive, and the factors underlying incomplete penetrance and variable expressivity are poorly understood. The yield of genetic screening is often low; for example, Mazzanti et al. reported a diagnostic rate of only 14%, despite familial disease being present in 44% of kindreds [23]. In this context, ECG screening in Family 1 identified two additional affected relatives despite negative genetic results (Figure 2), underscoring the fact that clinical evaluation remains essential, even in gene-elusive families.

According to ClinGen, only four genes have sufficient evidence for disease causation in SQTS and should be included in genetic testing panels for SQTS: *KCNH2* (definitive, autosomal dominant [AD] inheritance), *KCNQ1* (strong, AD), *KCNJ2* (strong, AD), and *SLC4A3* (moderate, autosomal recessive [AR] inheritance) [13]. Most evidence is based on a very limited number of variants, and several genes remain disputed (*SLC22A5*, *CACNA1C*, and *CACNA2D1*), with additional reports implicating sodium channel variants (*SCN5A* and *SCN10A*) or overlap with Brugada syndrome, though without a conclusive SQTS diagnosis [3,13].

In our genetic panel, we include the four validated SQTS genes, plus two disputed genes (all except *SLC22A5*) and *SCN5A*, to maximize diagnostic yield. Family history, genetic counseling, and combined genetic and clinical screening are essential for risk stratification [3,24]. Genetic testing is advised for diagnostic confirmation as well as family evaluation [10]. Nevertheless, only pathogenic or likely pathogenic variants in recognized genes should guide clinical practice, given the rarity of the disease. In addition, even when pathogenic variants are identified, genotype–phenotype correlations remain incomplete, and clinical expressivity is highly variable. In SQTS, clinical management should primarily follow the phenotypic diagnosis, whereas genotype-positive relatives without a definite phenotype require individualized follow-up rather than automatic classification as SQTS patients. Family 2 exemplifies incomplete penetrance, with a female carrier of the pathogenic *KCNJ2* variant exhibiting a negative phenotype (QTc > 360 ms), possibly reflecting estrogen-mediated QT prolongation in women [25].

Risk stratification in SQTS remains a major clinical challenge. Due to the rarity of the condition and lack of prospective registries, evidence guiding management—particularly in asymptomatic individuals—is limited. ESC guideline recommendations are all level C, with a single Class I indication for ICD implantation in secondary prevention (aborted cardiac arrest or documented sustained ventricular tachycardia) [10]. Routine prophylactic ICD implantation in asymptomatic patients is not supported [4,26]. Observational studies indicate a low incidence of appropriate ICD therapies in asymptomatic SQTS patients without prior syncope, ventricular arrhythmia, or cardiac arrest [26,27]. Consequently, the potential risks of interventions, including ICD implantation or antiarrhythmic drug therapy, often outweigh the uncertain benefits in this population [26,27]. ESC guidelines suggest that ICD should be considered in patients with arrhythmic syncope [10]. In our cohort, none of the patients experienced cardiac arrest, arrhythmic syncope, or spontaneous sustained ventricular tachycardia. However, in Family 1, ICD implantation was considered on an individualized basis, based on the Class IIb recommendation [10], because of the combination of a short QT phenotype and a strong family history of premature sudden cardiac death, after multidisciplinary review and shared decision-making. This should be interpreted as a case-specific preventive decision rather than a general recommendation for asymptomatic SQTS carriers.

Regarding pharmacological therapy, quinidine is the only antiarrhythmic with demonstrated efficacy in prolonging QT and suppressing arrhythmias, particularly in *KCNH2*-related forms [26,28,29]. However, its use in asymptomatic patients is controversial due to the risk–benefit profile, potential adverse effects, including gastrointestinal intolerance, proarrhythmic profile, cinchonism, thrombocytopenia, and hepatotoxicity [28,30]. Other antiarrhythmics (sotalol, flecainide, and ibutilide) are generally ineffective [28] and QT-shortening drugs, such as nicorandil, should be avoided [31]. Isoprenaline may be used in electric storm management but not for prevention [10].

Accordingly, the phenotype-positive individuals and genotype-positive relatives in both families were managed conservatively, with periodic ECG monitoring and clinical evaluation. No arrhythmia was documented during follow-up, and pharmacologic therapy was not initiated. Implantable loop recorder placement was considered in the asymptomatic child [10].

## 5. Limitations

This study has several limitations. Findings should be considered exploratory and hypothesis-generating. First, it is a retrospective review of only two families, reflecting the rarity of SQTS and limiting the generalizability of the findings. Additionally, the study population was derived from a referral-based cohort of patients evaluated for suspected inherited arrhythmia syndromes, which introduces potential selection and referral bias. Therefore, these findings should not be interpreted as representative of disease prevalence or risk in the general population. We also acknowledge that retrospective application of ESC criteria may introduce selection bias. Second, the duration of follow-up may not have been sufficient to capture late-onset arrhythmic events. Third, the family history of sudden death lacks autopsy confirmation, precluding definitive determination of the cause of death. Finally, as with all observational studies, management decisions were influenced by local practices, multidisciplinary consensus, and shared decision-making, which may differ in other centers.

## 6. Conclusions

In this national reference-center review, two families with a diagnosis of short QT syndrome were identified: one genetically negative family with phenotype-positive affected members, and one *KCNJ2*-positive family with a phenotype-positive proband and a genotype-positive/phenotype-negative relative. All individuals showed stable QT intervals (none < 320 ms) and there were no malignant arrhythmic events during follow-up. These findings highlight the rarity and heterogeneity of SQTS, the importance of both genetic and clinical screening and the ongoing uncertainty surrounding risk stratification. Systematic follow-up and inclusion in multicenter registries are crucial to improve understanding of this condition and support evidence-based management.

## Figures and Tables

**Table 1:** Summary of phenotype and/or genotype-positive SQTS individuals.

	Family 1			Family 2	
	Proband (II.3)	Sister (II.2)	Nephew (III.1)	Proband (III.1)	Mother (II.2)
Genetic results	Negative	Negative	Negative	*KCNJ2* p.Asp172Asn	*KCNJ2* p.Asp172Asn
QTc (ms) by:	<360	<360	<360	<360	>360
-Bazett;	334	336	334	341	365
-Fridericia;	337	333	336	330	375
-Framingham;	335	335	335	336	369
-Hodges;	338	333	337	332	380
Cardiac rate.	57 bpm	63 bpm	58 bpm	73 bpm	51 bpm
Classification	Phenotype-positive	Phenotype-positive	Phenotype-positive	Genotype-positive/phenotype-negative	Genotype-positive/phenotype-negative
Family history of SD	Yes	Yes	Yes	No	No
Syncope	No	No	No	No	No
AF	Yes	No	No	No	No
NSVT	No	No	No	No	No
Preventive treatment	ICD	No	No	No	No
Structural heart disease	No	No	No	Muscular VSD	No
Symptoms during follow-up	No	No	No	No	No

Phenotype-positive: individuals fulfilling ESC diagnostic criteria for SQTS. Genotype-positive/phenotype-negative: variant carriers without a clinical SQTS phenotype. AF: atrial fibrillation, BPM: beats per minute, NSVT: non-sustained ventricular tachycardia, SC: sudden death; VSD: ventricular septal defect. Data are descriptive and not intended to infer arrhythmic risk.

**Table 2:** Classification of *KCNJ2* (NM_000891.3):c.514G>A p.(Asp172Asn), according to ACMG/AMP criteria [19].

Criterion	ACMG/AMP Criterion	Given Strength	Justification
PS3	Well-established in vitro or in vivo functional studies supportive of a damaging effect on the gene or gene product	Strong	Functional studies using 2D ventricular cell and 3D tissue models revealed a significant increase in the outward component of the I-V relation of I(K1) and an increasing susceptibility to arrhythmia [22]
PM1	Located in a mutational hot spot and/or critical and well-established functional domain (e.g., active site of an enzyme) without benign variation	Moderate	27 pathogenic or likely pathogenic reported variants were found in a 419 bp region surrounding this variant in exon 2 within the region 68171533–68171952 without any missense benign variants
PM2	Absent from population databases	Moderate	The variant is absent from large population databases (rs104894584)
PP1	Co-segregation with disease in multiple affected family members	Supporting	The variant segregates with disease in a family with 2 affected individuals, while all unaffected family members are negative for this variant [21]
PP2	Missense variant in a gene that has a low rate of benign missense variation and in which missense variants are a common mechanism of disease	Supporting	Missense variant in a gene with low rate of benign missense variants for which missense variants are a common mechanism of a disease
PP3	Multiple lines of computational evidence support a deleterious effect	Supporting	In silico tools consistently predict that the variant disrupts the canonical splice acceptor site, leading to abnormal splicing (e.g., BDGP and SpliceAI), fulfilling PP3
PP5	Reputable source recently reports variant as pathogenic	Supporting	This variant has been classified as Pathogenic by 3 submissions in ClinVar (https://www.ncbi.nlm.nih.gov/clinvar/variation/8927/ accessed on 21 October 2025)

## Data Availability

The data presented in this study are not publicly available due to patient privacy and ethical restrictions. De-identified data may be made available from the corresponding author upon reasonable request and following institutional data-sharing agreements.

## Abbreviations

SQTS	Short QT Syndrome
ESC	European Society of Cardiology
ICD	Implantable Cardioverter-Defibrillator
